# Myocardial Infarction after Long-Term Treatment with a Tyrosine Kinase Inhibitor (TKI) with Anti-VEGF Receptor Activity

**DOI:** 10.1155/2019/7927450

**Published:** 2019-06-10

**Authors:** L. Paschke, T. Lincke, K. Mühlberg, Tom H. Lindner, R. Paschke

**Affiliations:** ^1^Division of Endocrinology, Department of Endocrinology and Nephrology, University Clinic Leipzig, Germany; ^2^Division of Nuclear Medicine, Department of Radiology, University Clinic Leipzig, Germany; ^3^Department of Angiology, University Clinic Leipzig, Germany; ^4^Division of Nephrology, Department of Endocrinology and Nephrology, University Clinic Leipzig, Germany; ^5^Department of Medicine, Division of Endocrinology, Departments of Oncology, Pathology, and Biochemistry and Molecular Biology & Arnie Charbonneau Cancer Institute Cumming School of Medicine, University of Calgary, Canada

## Abstract

TKIs including anti-VEGF receptor activity have been approved for the treatment of patients with radioiodine resistant thyroid carcinomas. For lenvatinib arterial thromboembolic events are listed as adverse events of special interest with lenvatinib. In the phase III study, arterial thromboembolic events were reported in 3% of lenvatinib-treated patients and 1% in the placebo group. Most of the patients had predisposing factors. Only one myocardial infarct was reported in the lenvatinib phase III study. We report a 73-year-old female patient with metastatic thyroid papillary carcinoma who was treated with total thyroidectomy. The operation was followed by four radioiodine therapies over a period of 6 years. At 6 years she developed lung metastasis without radioiodine uptake, one solitary liver metastasis and one solitary right renal metastasis. One year after the first diagnosis of radioiodine resistant lung metastasis the lung metastasis showed progression according to RECIST criteria. This treatment was resulting in prolonged partial response with disappearance of a hepatic and renal metastasis. A myocardial infarction occurred after 39 months of lenvatinib treatment resulting in implantation of 3 stents and a two chamber pacemaker. The treatment was discontinued. Except for well controlled hypertension there were neither predisposing diseases like diabetes nor symptoms of cardiac ischemia on exertion. However, the family history for cardiovascular diseases was positive for cardiac infarction reported for one brother. Another brother was treated for hypertension and the patient's mother suffered from a cerebral infarction at the age of 60. While only one myocardial infarct was reported in the lenvatinib phase III study with 392 patients this case suggests that long-term treatment with lenvatinib may be associated with an increased risk for myocardial infarct also in patients with no predisposing diseases except well controlled hypertension and positive family history for cardiovascular diseases.

## 1. Introduction

In 2015 lenvatinib, an inhibitor of vascular endothelial growth factor receptors 1, 2, and 3, fibroblast growth factor receptors 1 through 4, platelet-derived growth factor receptor *α*, RET, and KIT signaling networks [1, 2], has been approved by the FDA and EMA for the treatment of radioiodine refractory differentiated thyroid cancer.

A randomized, double-blinded phase III study involved patients with progressive thyroid cancer that was refractory to ^131^iodine and randomly assigned 261 patients to receive lenvatinib (daily dose of 24 mg/d in 28-day cycles) and 131 patients to receive placebo. Compared with placebo, lenvatinib was associated with significant improvements in progression-free survival and the response rate among patients with ^131^iodine-refractory thyroid cancer [3]. During the 13.8 months of median duration of treatment, treatment-related adverse events (all grades) occurred in 97.3% in the lenvatinib group; of these 75.9% were treatment-related adverse events of grade 3 or higher [3]. In the SELECT trial 3.0% of lenvatinib-treated patients presented arterial thromboembolic events. Most of the patients had cardiovascular risk factors. Only one myocardial infarct was reported.

Here, we report a patient with papillary thyroid cancer who suffered from a myocardial infarction during long-term treatment with lenvatinib. This case cautions that long-term treatment with lenvatinib can be associated with myocardial infarction also in patients who were asymptomatic and had no predisposing diseases except well controlled hypertension and a positive family history for cardiovascular diseases.

## 2. Patient

We report a 73-year-old female patient with metastatic thyroid papillary carcinoma who was treated with total thyroidectomy. The operation was followed by four radioiodine therapies over a period of 6 years. At 6 years she developed lung metastasis without radioiodine uptake, one solitary liver metastasis and one solitary right renal metastasis. One year after the first diagnosis of radioiodine resistant lung metastasis the lung metastasis showed progression according to RECIST criteria. The patient was therefore enrolled in the phase III study comparing lenvatinib to placebo. After the study ended the patient was unblinded. Lenvatinib treatment resulted in prolonged partial response with disappearance of the hepatic and renal metastasis. During further treatment with lenvatinib with dose reduction from initially 24 to 10 mg at 17 months of lenvatinib treatment a myocardial infarction occurred after 39 months of lenvatinib treatment resulting in implantation of 3 stents and a two chamber pacemaker. Treatment with lenvatinib was discontinued at the time of diagnosis of the myocardial infarction. Except for well controlled hypertension there were neither predisposing diseases like diabetes nor symptoms of cardiac ischemia on exertion. Quarterly repeated echocardiography at rest showed normal results during the first two years of lenvatinib treatment during the phase III study. However, the family history for cardiovascular diseases was positive for cardiac infarction reported for one brother. Another brother was treated for hypertension and the patients' mother suffered from a cerebral infarction at the age of 60.

## 3. Discussion

Tyrosine kinase inhibitors with anti-VEGF receptor activity are known for their potential to cause thromboembolic adverse events.

Sorafenib, an inhibitor of VEGFR-1, VEGFR-2, and VEGFR-3, RET (including RET/PTC translocations), RAF (including BRAFV600E point mutation), and platelet-derived growth factor receptor beta (PDGFR*β*) [4], was approved for the treatment of radioiodine refractory DTC in 2014 by the EMA and FDA. Before it was also approved for the treatment of unresectable hepatocellular carcinoma and advanced renal-cell carcinoma.

A randomized, double-blind, placebo-controlled phase III study involving patients with advanced clear-cell renal-cell carcinoma reported cardiac ischemia or infarction in 12 patients in the sorafenib group (3%) and 2 patients in the placebo group (<1%) (P=0.01). Of these events, 11 (including 2 deaths in the sorafenib group and 1 death in the placebo group) were considered to be serious adverse events associated with treatment [5]. A phase 3, double-blinded, placebo-controlled study assigned 602 patients with advanced hepatocellular carcinoma who had not received previous systemic treatment to receive either sorafenib (at a dose of 400 mg twice daily) or placebo. Cardiac Ischemia or infarction appeared in 3% of the patients receiving sorafenib compared to 1% in the placebo group [6]. The median durations of sorafenib treatments in these studies were 10.6 months [4], 5.8 months [5], and 5.3 months [6].

Furthermore, soon after the introduction of bevacizumab-treatment for metastatic breast cancer the clinical incidence of arteriovascular events, i.e., cerebrovascular ischemia was reported in 2% of the patients and 1.6% of the patients suffering from heart failure and even more alarming 15% having severe hypertension [7]. In addition, meta-analysis showed that the addition of bevacicumab to chemotherapy increased the risk of arterial thrombotic events when compared to the chemotherapy alone [8]. Moreover a pooled analysis of five randomized trials encompassing 1745 patients randomly assigned to chemotherapy alone or chemotherapy plus bevacicumab for the treatment of metastatic colorectal cancer, breast cancer, and non-small-cell lung cancer showed that the increase of arterial thromboembolic events (ATEs) was further increased in elderly patients or patients with a history of ATEs. Patients with both risk factors developed ATEs in 17.9% [9].

A meta-analysis including 9711 patients from 19 randomized controlled trials with bevacicumab [10] revealed an overall incidence of ATEs of 1,5% whereof cardiac ischemia (67,4%), CNS ischemia (7,9%), and cerebrovascular accident (6,7%) were most common. The mortality of these ATEs was 8,2% [10].

Because of the thromboembolic potential of TKIs Conti et al. proposed in 2013 a cardiovascular assessment of patients undergoing a TKI treatment before treatment start and to include patients at high/highest risk according to established risk scores like the Global Registry of Acute Coronary Events (GRACE) score in a cardiovascular monitoring program [11].

Recently, a multicenter phase 2 study of sunitinib another antiangiogenic tyrosine kinase inhibitor in patients with locally advanced or metastatic differentiated, anaplastic or medullary thyroid carcinomas, revealed that during a median follow-up time of 9 months/25 months 14.1% of the patients suffered from cardiac events [12]. Out of 5 deadly adverse events 4 were cardiac events. Due to an expected increased risk of severe adverse cardiac events the follow-up of the study included systematic survey, a left ventricular ejection fraction (LVEF) ultrasound, and N-terminal probrain natriuretic peptide or N-terminal brain natriuretic peptide (probrain natriuretic peptide [BNP]/BNP) dosage. None of these specific parameters showed the potential to select patients more stringently [12].

Furthermore a meta-analysis on 4679 patients under treatment with sorafenib or sunitinib or pazopanib demonstrated an increased risk of fatal adverse events (RR 2.23) regardless of tumor type and drug used, when compared with control groups. Myocardial infarction represented the cause in 15% of all deaths attributable to VEGFR inhibitor [13].

Our case report suggests that lenvatinib should also be added to the list of TKIs with ATE potential. So far, most of the thromboembolic events appeared after short-term treatment median duration of 10.8 months with tyrosine kinase inhibitors and mostly in patients with predisposing factors. However, our patient suffered from her first myocardial infarction after long-term treatment with lenvatinib for 39 months, in absence of predisposing diseases except well controlled hypertension and a positive family history for cardiovascular diseases. These findings suggest that long-term treatment with lenvatinib may be associated with an increased risk for myocardial infarction. Therefore, as previously proposed by Conti et al. for other TKIs also patients with lenvatinib treatment should be assessed for cardiovascular risk and coronary ischemia before and during the treatment. However, as indicated by our patient not only patients with high/very high cardiovascular risk factors should be under closer clinical cardiovascular surveillance as our patient's GRACE score before study entry indicated only a low individual risk of death by myocardial infarction. The assessment of cardiovascular risk and coronary ischemia should include a family history for cardiovascular diseases, cardiac stress testing, and an initial and regular cardiac survey to identify those at increased risk for cardiac events. It should be performed before starting lenvatinib treatment and at annual intervals during lenvatinib therapy. In addition, further data on adverse events during long-term TKI treatment with anti-VEGF receptor activity should be systematically collected.
